# Face Anti-Spoofing Based on Adaptive Channel Enhancement and Intra-Class Constraint

**DOI:** 10.3390/jimaging11040116

**Published:** 2025-04-10

**Authors:** Ye Li, Wenzhe Sun, Zuhe Li, Xiang Guo

**Affiliations:** School of Computer Science and Technology, Zhengzhou University of Light Industry, Zhengzhou 450002, China; acm@zzuli.edu.cn (Y.L.); zuheli@zzuli.edu.cn (Z.L.); 332305090552@zzuli.edu.cn (X.G.)

**Keywords:** face anti-spoofing, enhanced channel attention, intra-class differentiator

## Abstract

Face anti-spoofing detection is crucial for identity verification and security monitoring. However, existing single-modal models struggle with feature extraction under complex lighting conditions and background variations. Moreover, the feature distributions of live and spoofed samples often overlap, resulting in suboptimal classification performance. To address these issues, we propose a jointly optimized framework integrating the Enhanced Channel Attention (ECA) mechanism and the Intra-Class Differentiator (ICD). The ECA module extracts features through deep convolution, while the Bottleneck Reconstruction Module (BRM) employs a channel compression–expansion mechanism to refine spatial feature selection. Furthermore, the channel attention mechanism enhances key channel representation. Meanwhile, the ICD mechanism enforces intra-class compactness and inter-class separability, optimizing feature distribution both within and across classes, thereby improving feature learning and generalization performance. Experimental results show that our framework achieves average classification error rates (ACERs) of 2.45%, 1.16%, 1.74%, and 2.17% on the CASIA-SURF, CASIA-SURF CeFA, CASIA-FASD, and OULU-NPU datasets, outperforming existing methods.

## 1. Introduction

Face detection technology has found widespread application in areas such as security surveillance and smart device unlocking, with its accuracy and efficiency significantly enhanced by the adoption of deep learning techniques, particularly Convolutional Neural Networks (CNNs). In financial transactions and security systems, face anti-spoofing technologies are especially critical for preventing unauthorized access and identity theft, thereby safeguarding personal privacy and maintaining social order. However, current face anti-spoofing technologies still face various forms of spoofing attacks, necessitating the development of simple, yet efficient, methods to enhance their security and reliability.

There are three primary types of spoofing attacks against face recognition systems: photo, video replay, and 3D mask attacks. A photo attack occurs when an attacker uses a printed photograph of a legitimate user to deceive the face recognition system. A video replay attack involves an intruder using a video of the legitimate user to trick the system. 3D mask attacks, which involve the use of high-quality masks that closely mimic the facial features of the legitimate user, further complicate detection. The diversity and complexity of these attack methods highlight the pressing need for robust face anti-spoofing measures.

Deep learning has made significant advancements in single-modal facial liveness detection. However, existing face anti-spoofing detection methods still struggle to handle increasingly complex spoofing attacks and adapt to diverse environments. Under extreme variations in lighting intensity and angle, it is hard to effectively integrate local and global features and capture subtle spoofing cues. This often leads to a significant drop in detection accuracy. Additionally, the feature space exhibits significant overlap between live and spoofed samples, making it difficult for the model to distinguish them accurately and increasing the difficulty of classification. Researchers have proposed various methods to address these challenges, such as Arman Keresh [1] using a multi-head self-attention mechanism to capture local and global correlations in images. Almeida [2] enhances the robustness of the model by using a patch-based CNN and multi-objective loss function. William [3] combines low-level features and partial least squares regression (PLS) to enhance detection accuracy. Wang [4] proposed comparing depth loss to accurately supervise depth features. Liu [5] proposed FM-ViT, which processes different modal features through dedicated branches and introduces the CMTB module to extract latent features and learn cross-modal active features. Liao et al. [6] proposed the Domain-Invariant Vision Transformer (DiVT) and incorporated both concentration loss and separation loss to learn domain-invariant feature representations, thereby improving the generalization ability of face anti-spoofing models in unknown domains. Liu et al. [7] proposed the Modality-Agnostic Vision Transformer (MA-ViT), which significantly improves single-modal face anti-spoofing performance through early fusion of multi-modal data and by designing the Modality-Disentangle Attention (MDA) and Cross-Modal Attention (CMA) within the Modality-Agnostic Transformer Block (MATB).

Combining the results and benefits of the aforementioned research, we propose a method based on the Enhanced Channel Attention (ECA) module and the Intra-Class Differentiator (ICD). The ECA module integrates deep convolution with the BRM, extracting spatial features for each channel independently while preserving local information. It also enhances key feature extraction via a hierarchical information redistribution mechanism. The ICD module reduces intra-class feature dispersion by enforcing compactness constraints. It also maximizes the feature distance between live and spoofed samples through separation constraints, thereby optimizing the classification boundary. This unique design not only enhances the model’s stability under varying lighting conditions and complex backgrounds but also significantly improves the accuracy of live face detection. Our contributions are as follows:

(1) We propose a novel face anti-spoofing detection framework that integrates a dual strategy of feature enhancement and constraint optimization. This framework incorporates the Enhanced Channel Attention (ECA) module and the Intra-Class Differentiator (ICD) module, which dynamically adjust the importance of channel features and optimize sample distribution in the feature space, thereby enhancing the model’s discriminative power.
(2) The ECA module integrates deep convolution with the Bottleneck Reconstruction Module (BRM) and a channel attention mechanism. By leveraging deep convolution to extract local spatial features and refining channel features through the BRM, the ECA module further enhances the representation of critical channels. The attention mechanism dynamically adjusts feature importance, enabling the model to distinguish live and spoofed samples more accurately.
(3) The ICD module introduces intra-class compactness constraints and inter-class separability constraints to dynamically optimize sample distribution in the feature space. By minimizing intra-class variations and maximizing inter-class feature distances, the ICD module effectively refines classification boundaries. This significantly enhances the clustering of live samples and improves the model’s discriminative ability for spoofed samples.

The structure of this paper is as follows. Section 2 reviews related research in the field of face anti-spoofing detection. Section 3 provides a detailed introduction to our proposed model. Section 4 describes the used datasets, evaluation methods, and experimental details. Section 5 presents the experimental results. Section 6 summarizes the main contributions of our research, discusses existing limitations, and proposes future research directions.

## 2. Related Works

### 2.1. Face Anti-Spoofing

Over the past decade, research in the field of live face detection has surged, primarily driven by the need to counteract face presentation attacks [8]. Traditional methods often rely on handcrafted features, which demonstrate varying degrees of effectiveness under specific conditions but also exhibit notable limitations. Pan et al. [9] tackled the face liveness detection task using handcrafted features; however, their approach’s heavy dependence on feature design led to poor performance in complex scenarios. Patel et al. [10] demonstrated that the Scale-Invariant Feature Transform (SIFT) descriptor exhibits robustness, particularly under scale variations, lighting conditions, and local affine distortions. Nevertheless, the high computational complexity of SIFT renders it unsuitable for real-time applications. Similarly, Komulainen et al. [11] employed the Histogram of Oriented Gradients (HOG) method to achieve satisfactory detection results on single frames and video sequences. However, HOG’s limited capacity to process global image information hampers its ability to capture fine-grained features in complex backgrounds.

Freitas et al. [12] proposed incorporating spatial and temporal information through Local Binary Patterns (LBPs) to enhance model discriminative ability. Although these methods improved the detection of face spoofing to some extent, their robustness remained constrained by illumination variations and the complexity of background settings. Boulkenafet et al. [13] proposed describing face appearances using accelerated robust features across different color spaces to address spoofing attacks. However, the generalization ability of this method in multimodal scenarios remains limited. While handcrafted features in early studies offered valuable insights for liveness detection tasks, their inherent limitations are evident. These approaches typically rely on predefined rules, lacking adaptability to complex backgrounds, dynamic lighting, and diverse spoofing techniques.

### 2.2. FAS Based on Global Feature Fusion

With the advent of deep learning, an increasing number of researchers have adopted deep learning methods for face anti-spoofing detection. Among these studies, some focus on extracting global features from RGB images for analysis [14]. Menotti et al. [15] employed Convolutional Neural Networks (CNNs) to extract spoofing features, using Support Vector Machines (SVM) as classifiers. This approach initially demonstrated the potential of CNNs in feature extraction but showed limited adaptability to complex lighting and diverse environmental conditions. To further enhance feature extraction capabilities, Wang et al. [16] proposed a model combining local Convolutional Neural Networks with a global Multilayer Perceptron (CONVN-MLP) fusion. This model applies convolution operations on image patches to extract local features and utilizes cross-patch mixing techniques to capture global dependencies between patches. Although this approach effectively compensated for the limitations of local feature extraction, the stability of its feature representation remained challenging in complex settings.

Wang et al. [17] proposed that when using complete face images for training, neural networks may overlook certain detailed information. To address this issue, they divided face images into multiple smaller regions, with the aim of capturing richer local features. While this strategy enhanced the ability to capture local information to some extent, the segmentation approach introduces additional computational costs and does not fully resolve the problem of global dependencies. In response to these challenges, many studies have begun incorporating attention mechanisms to enhance feature extraction capabilities. For example, Hu et al. [18] proposed the SE module, which compresses feature maps into channel-wise descriptors using global average pooling. However, this process completely discards spatial information, making it difficult for the SE module to capture spoofing-related spatial features in face images effectively. As a result, it struggles to leverage spatial information for liveness detection. Subsequent improvements, such as CBAM [19] and BAM [20], introduced spatial attention mechanisms to enhance spatial feature extraction. However, these methods still have limitations in optimizing dynamic relationships between channels. While CBAM uses a multi-layer perceptron to learn channel-wise weights, its ability to model dynamic inter-channel dependencies remains limited. Similarly, BAM’s channel attention mechanism relies solely on global average pooling to generate channel weights, lacking complex inter-channel interactions. This makes it difficult to capture subtle spoofing cues in face images, ultimately restricting its effectiveness in face anti-spoofing tasks.

With the application of Transformer architectures to visual tasks, Dosovitskiy et al. [21] introduced the Vision Transformer (ViT) network structure, which processes images by dividing them into sequences of patches and employing multi-head self-attention mechanisms to handle long-range dependencies. This approach offers a highly flexible framework for image representation. However, ViT still exhibits limitations in feature integration and channel enhancement, particularly in complex scenarios, where it struggles to effectively capture critical deceptive features. George et al. [22] were the first to adapt the ViT architecture for face detection tasks, optimizing its performance on real-time detection benchmarks through parameter adjustments.

### 2.3. Feature Representation Learning

To more accurately and efficiently capture the characteristics of face spoofing behaviors, many researchers have adopted feature representation learning methods, enabling models to extract more discriminative information. However, in the task of face anti-spoofing, visual facial features are often accompanied by background noise and other confounding factors. Consequently, enhancing the robustness of models to these disturbances remains a critical challenge in the field of single-modal face anti-spoofing research.

Chen et al. [23] designed a binary focal loss function that focuses on distinguishing between easy and hard samples. By expanding the feature disparity between genuine and spoof faces, this approach enhances the network’s ability to recognize different spoofing techniques. However, this method faces the issue of feature distribution overlap in complex scenarios, making it difficult to correctly classify certain spoofed samples. George and Marcel [24] developed a method that combines a class contrastive loss with binary cross-entropy loss, forming a contrastive constraint mechanism. This mechanism performs excellently in adjusting sample distribution and enhancing feature discriminability; however, it still exhibits limitations when dealing with intra-class feature dispersion and complex backgrounds. Wang et al. [25] designed a dense similarity loss, which maximizes the similarity between intermediate features through a self-supervised approach. However, the effectiveness of this method heavily relies on the uniformity of the data distribution, significantly degrading when faced with imbalanced data or uneven sample distributions. Li et al. [26] employed graph nodes to establish semantic-aware node affinities and used graph edges as secondary constraints in the structurally aware matching loss, achieving fine-grained adaptivity through node-to-node graph matching. Yue et al. [27] proposed CTRL, a method that trains a neural network and clustering algorithm to detect label errors in multi-class datasets, removing noisy samples and retraining the model. Experimental results demonstrate its state-of-the-art accuracy on image and tabular data, with theoretical analysis supporting its superior performance. Dai et al. [28] introduced clustering contrast, calculating contrastive loss at the clustering level and incorporating momentum updates to enhance feature consistency.

Almeida et al. [2] employed triplet loss on RGB face data and incorporated sensor-aware loss to increase the separation between samples. Jiang et al. [29] proposed a parallel domain structure coupled with a regularization strategy to mitigate the adverse effects of limited sample size and the diversity of spoofed samples on conditional distribution alignment. However, the adaptability of this approach to different modalities and sample conditions requires further validation. Li et al. [30] introduced the U-KAN architecture, which integrates Kolmogorov–Arnold Networks (KANs) to enhance the performance and interpretability of U-Net through non-linear learnable activation functions, achieving higher accuracy and lower computational costs in image segmentation and diffusion models. Yang et al. [31] leveraged the automatic feature extraction capabilities of Convolutional Neural Networks (CNNs) and employed a binary Softmax loss function for supervised learning, yielding notable results. Nevertheless, this method still exhibits limitations in handling intra-class sample variations and ensuring the robustness of feature extraction.

In summary, existing methods struggle to capture critical spoofing cues in face images and often exhibit weak generalization ability. Moreover, most approaches rely on implicitly optimizing the relative distance between samples, making it difficult to directly constrain feature space distribution. This results in overlapping distributions of different classes and dispersed features within the same class, especially in complex scenarios. To address these limitations, our proposed ECA module independently extracts spatial features for each channel using deep convolution, preserving the local contextual information lost in traditional attention mechanisms. Additionally, it optimizes inter-channel dynamic relationships through a hierarchical information redistribution mechanism, enabling the model to capture key spoofing cues and improving its generalization ability. Furthermore, our ICD feature constraint enforces compact intra-class distributions to reduce feature dispersion while maximizing the feature distance between different classes. This effectively mitigates class overlap in feature space, enhancing the model’s classification accuracy.

## 3. Method

### 3.1. The Overall Structure

**Figure 1:**
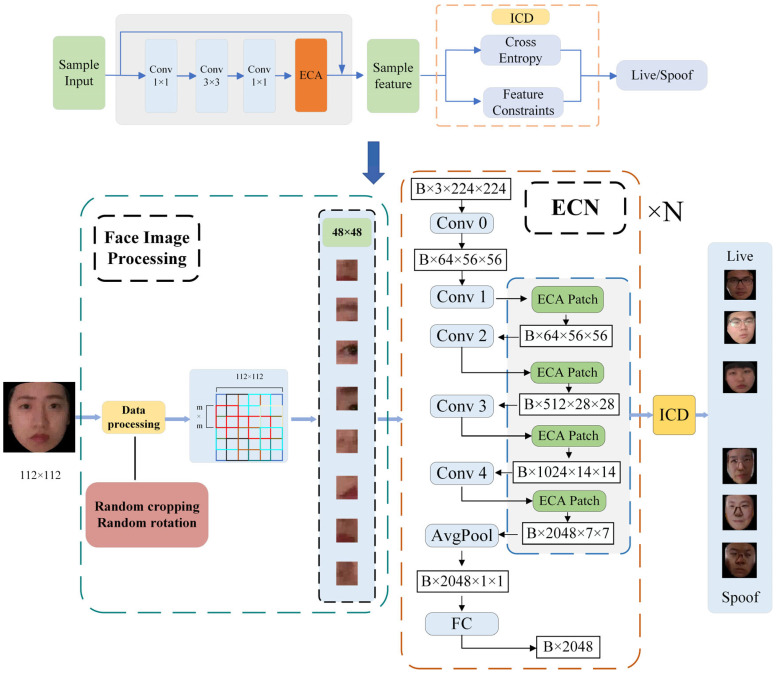
Overall framework structure diagram. The backbone network consists of ResNeXt and Enhanced Channel Attention (ECA), which enhances features through deep convolution and the BRM. The Intra-Class Differentiator (ICD) optimizes the network through feature constraints.

As shown in Figure 1, in order to effectively extract facial features, we added an ECA module to the network, which mainly consists of five convolution modules and four ECA modules. During the training process, the input face images are first randomly cropped and inverted to enhance data diversity. Then, the images are cropped to the desired size and sent to the network for feature extraction. Finally, the ECA module and ICD module are jointly optimized to guide the network to focus on learning and deception related characteristics.

### 3.2. The Enhanced Channel Attention ResNeXt Structure

**Figure 2:**
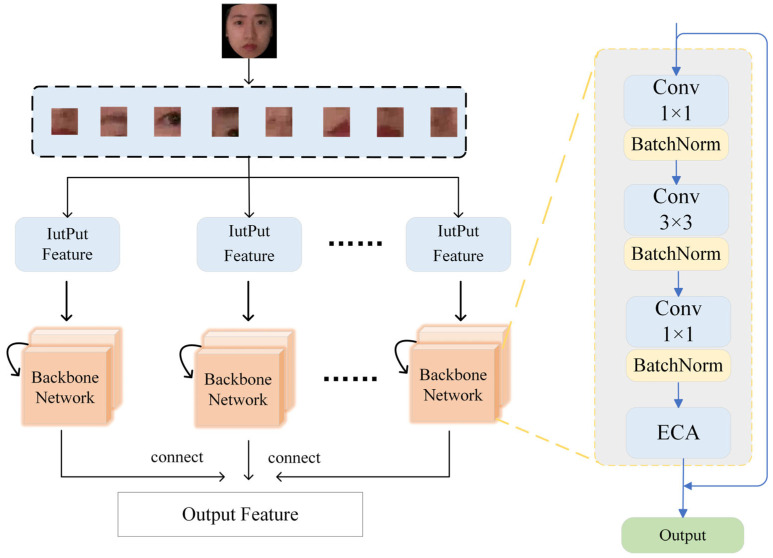
ResNeXt network structure with ECA module.

The ECA module is built upon the ResNeXt-50 network and is embedded into its grouped convolution structure. As shown in Figure 2, the feature extraction layer consists of multiple basic network blocks. Each block comprises a 1 × 1 convolution, a 3 × 3 convolution, another 1 × 1 convolution, and the ECA module. Additionally, residual connections are introduced to mitigate the vanishing gradient problem, ensuring more stable training. Table 1 shows the number of layers, convolution kernel size, output feature channel size, and stride size of the network architecture.

**Figure 3:**
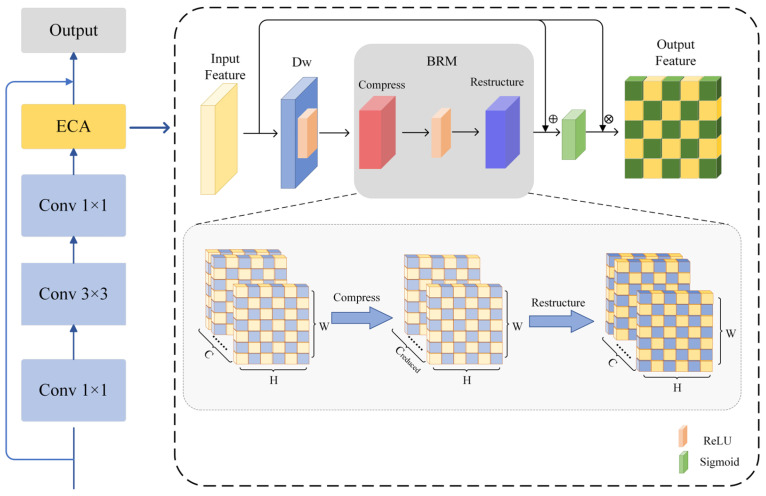
Flowsheet of the ECA module.

As shown in Figure 3, the feature map $X\in {\mathbb{R}}^{C\times H\times W}$ is input where *C* represents the number of channels, *H* and *W* represent the height and width of the feature map. The specific implementation steps of the ECA module are as follows: the input feature map X is first processed by the deep convolutional layer *K_Dw_*, whose formula is:

(1) $$X_{Dw}(c,i,j)=\mathrm{ReLU}\left(\sum_{p=0}^{k-1}\sum_{q=0}^{k-1}K_{Dw}(c,p,q)\cdot X(c,i+p,j+q)\right)$$

In this process, each channel *c* undergoes convolution independently; *i* and *j* represent the position indices in the output feature map *X_Dw_*. The weight matrix $K_{Dw}\in {\mathbb{R}}^{C\times k\times k}$ slides only within a single channel, where *K_Dw_*(*c*, *p*, *q*) denotes the weight of the depthwise convolution kernel at channel *c*, row offset *p*, and column offset *q*, and *X*(*c*, *i* + *p*, *j* + *q*) represents the value at channel *c*, row *i* + *p*, and column *j* + *q* of the input feature map. Subsequently, the output feature map *X_Dw_* is processed through the BRM module for feature reconstruction and channel-wise adjustment. The formula for the BRM is as follows:

(2) $$X_{reduced}=U_{c1}\Lambda_{c1}{V_{c1}}^{T}X_{Dw}$$

(3) $${X^{\prime }}_{reduced}=\sigma (X_{reduced})$$

(4) $$X_{flat}=U_{c2}\Lambda_{c2}{V_{c2}}^{T}{X^{\prime }}_{reduced}$$

We apply Singular Value Decomposition (SVD) for dimensionality processing of the features, where *V^T^* is the right singular vector matrix, used for channel feature transformation. $V_{c1}^{T}\;$ is responsible for mapping the original features to a low-dimensional subspace, performing dimensionality reduction and extracting the principal feature components. $V_{c2}^{T}$ remaps the non-linearly transformed features back to the original high-dimensional channel space. *U_c_*_1_ is the left singular vector matrix corresponding to the new basis vectors during the dimensionality reduction, while *U_c_*_2_ is the left singular vector matrix during the upscaling, ensuring that the reduced features can be remapped back to the original high-dimensional channels. Λ*_c_*_1_ is the diagonal matrix in the dimensionality reduction phase, representing the importance of the channel features. After dimensionality reduction, the feature dimension is C_reduced_ × H × W, while Λ_c2_ is the diagonal matrix in the upscaling phase, ensuring that the most critical features are restored, and the dimensionality of the upgraded features is restored to C × H × W. σ is the ReLU non-linear activation function, which increases the non-linear expression ability of the model.

To prevent feature loss, we introduce residual connections and apply the Sigmoid activation function to constrain the weights W within the range (0, 1). This helps mitigate the risk of gradient vanishing or explosion:

(5) $$W=Sigmoid(X+\alpha X_{flat})$$

(6) $$\alpha =\frac{∥X{∥}_{F}}{∥X_{flat}{∥}_{F}+\epsilon }$$

Here, *α* represents the ratio of the channel-adaptive weight norm, which adjusts the contribution of *X_flat_* to *X*. The term ||∙||*_F_* denotes the Frobenius norm. ϵ is a small constant to prevent division by 0. When the *X_flat_* is too large, *α* approaches 0, suppressing the interference of reconstructed features on the residual, conversely, enhancing their contribution.

Multiply the channel attention weight *W* with the original feature map *X* to obtain the output feature *Y*:

(7) Y(c,i,j)=W(c)⋅X(c,i,j)

Among them, *c* represents the index of the channel, and *i* and *j* represent the spatial coordinates of the feature map. Finally, the output *Y* is the feature of the input feature map X after passing through the ECA module.

### 3.3. Feature Constraint

The ICD module consists of a cross-entropy loss function and a global-local feature contrastive loss, which optimizes the feature space distribution through feature constraints and enhances the model’s discriminative ability. The implementation steps of the ICD module are as follows: let the feature vector sets of positive and negative samples be F = ${\left\{f_{i}\right\}}_{i=1}^{n_{p}}$ and G = ${\left\{g_{j}\right\}}_{j=1}^{n_{n}}$, where *f_i_* and *g_j_* are both d-dimensional vectors, and *N_p_* and *N_n_* represent the numbers of positive and negative samples. The feature distance adopts Euclidean distance as the measurement standard, and the formula is as follows:

(8) $$D(f_{i},f_{j})=\sqrt{\sum_{k=1}^{d}{\left(f_{i}^{k}-f_{j}^{k}\right)}^{2}}$$

In order to further constrain the feature compactness of similar samples and the feature separability of heterogeneous samples, we calculate the average Euclidean distance of positive and negative sample pairs, respectively:

(9) $$dist_{pos}=\frac{1}{n_{p}(n_{p}-1)}\sum_{i=1}^{n_{p}}\sum_{j=i+1}^{n_{p}}D(f_{i},f_{j})$$

(10) $$dist_{neg}=\frac{1}{n_{n}(n_{n}-1)}\sum_{i=1}^{n_{n}}\sum_{j=i+1}^{n_{n}}D(g_{i},g_{j})$$

Among them, *dist_pos_* represents the average feature distance between positive samples; *dist_neg_* represents the average feature distance between negative samples.

To simultaneously optimize the compactness of intra-class samples and the separation of inter-class samples, we propose a Global–Local Feature Contrastive Loss (GL-FCL), which integrates the distance constraints of positive and negative samples. The formula is as follows:

(11) $$L_{dist\_all}=dist_{pos}-\lambda \cdot dist_{neg}+\gamma \cdot R(F,G)$$

Among them, *λ* is a hyperparameter used to adjust the weight of inter-class separability in the loss function, while γ controls the influence of the global feature distribution constraint. *R*(*F*, *G*) represents the global feature distribution regularization term, which optimizes the global distribution structure of the feature space and prevents local collapse.

By minimizing *dist_pos_*, we ensure that samples of intra-class are as close as possible in the feature space, thereby enhancing intra-class compactness. Simultaneously, we maximize *dist_neg_* to increase the distance between samples of inter-class. Additionally, by introducing the weighting factor *λ* and taking its negative value, the optimization process encourages the model to focus on enlarging the distance between different class samples, strengthening inter-class separability, and ultimately improving the model’s ability to distinguish between different categories of samples.

In practical optimization, relying solely on intra-class compactness and inter-class separability may lead to local contraction in the feature space, potentially causing the collapse of decision boundaries. To address this issue, we introduce a global feature distribution regularization term:

(12) $$R(F,G)=\sum_{k=1}^{d}{\left(\sigma_{F}^{k}-\sigma_{G}^{k}\right)}^{2}$$

where $\sigma_{F}^{k}$ and $\sigma_{G}^{k}$ represent the variances of positive and negative samples along the *k*-th dimension. These variances measure the distribution differences of positive and negative samples in the feature space. To further enhance the model’s classification performance, we combine the GL-FCL with the cross-entropy loss to form the ICD. The cross-entropy loss function is defined as

(13) $$L_{CE}=-[y_{i}\log(y_{j})+(1-y_{i})\log(1-y_{j})]$$

where *y_i_* represents the true label of the sample and *y_j_* denotes the probability predicted by the model. The final joint optimization objective function is as follows:

(14) $$L_{all}=\alpha \cdot L_{CE}+\beta \cdot L_{dist\_all}$$

where *α* and *β* are hyperparameters used to balance the influence between the cross-entropy loss and the GL-FCL.

**Figure 4:**
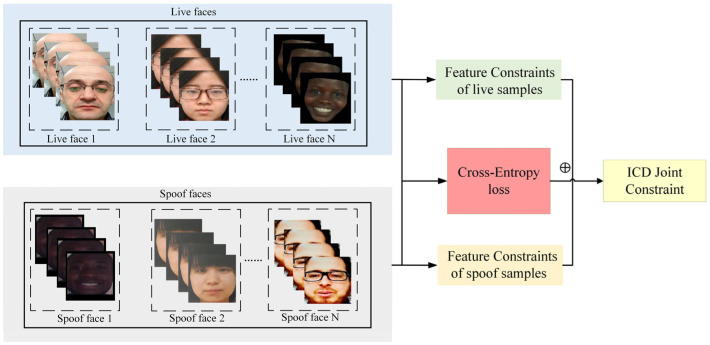
Schematic diagram of the overall joint feature constraint optimization framework.

As shown in Figure 4, We separately input live samples and spoofed samples into feature constraints, calculate the GL-FCL of the two types of samples, and calculate the cross-entropy loss of the two types of samples. Finally, we combine these two types of constraints to learn the deep features of face images. Through this approach, the ICD module not only optimizes the model’s feature learning ability but also enhances the model’s recognition of live features and anti-interference ability against spoofed samples.

**Figure 5:**
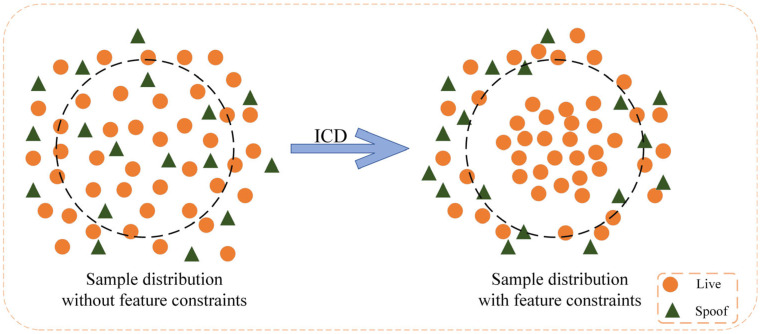
Intra-Class Differentiator (ICD) constraint effect diagram.

The effect of the ICD constraint is illustrated in Figure 5. Without the ICD constraint, the sample distribution is relatively dense, and the boundaries between different classes are not clearly defined. As a result, the classifier may struggle to distinguish between similar samples, leading to potential confusion. When the ICD constraint is introduced, the sample distribution is significantly optimized. The representation of live samples becomes more compact, bringing them closer to the center of the live sample class, while spoofed samples are penalized, pushing them away from the clustering center.

Finally, the entire algorithm process of our framework is shown in Algorithm 1.

**Algorithm 1:** Training Algorithm for the ECN Network Model
**Input:**
       **Dataset:** Mixed dataset containing live and spoofed samples.       **Model:** Network architecture with ECA and ICD modules.       **Optimizer: SGD** (momentum = 0.9, weight decay = 0.005).       **Hyperparameters:**
$\mathrm{Batch}\;\mathrm{size}=128,\;\mathrm{train}\;\mathrm{rounds}=10,\;\mathrm{epochs}\;\mathrm{per}\;\mathrm{round}=50,\;\mathrm{loss}\;\mathrm{weight}:\;𝛼=\;1,\;𝛽=\;1,\;\mathrm{Initial}\;\mathrm{learning}\;\mathrm{rate}:\;\eta_{\mathrm{init}}=1\times 10^{-3}.$**Output:** The trained model parameters ***Φ_F_***.
**Training Process:**

**1. Initialize Training**
         Set initial learning rate η_init_.
**2. Outer Training Loop (Rounds 1 to 10)**
    Decay learning rate: *η_t_* = *η_init_* × 0.95^round^.
**3. Inner Training Loop (Epochs 1 to 50)**
    Iterate through the train dataset in mini-batches.
**4. Batch Processing**
    a. Randomly shuffle live and spoofed samples.    b. Input live samples and compute the representation loss dist_Pos_ as (9)    c. Input spoofed samples and compute the representation loss dist_Neg_ as (10)    d. Compute the basic loss L_CE_ and constrained loss L_dist_all_ as (11) and (13)    e. Compute the overall loss L_all_ as (14)    f. Perform backpropagation and update the model parameters.
**5. Validation and Evaluation**
    - Evaluate the model on the validation set.    - Compute ACER.
**6. Repeat Steps 2–5 until all rounds are completed.**

**7. End of Training: The final model *Φ_F_* is obtained.**


## 4. Experiments

### 4.1. Datasets

We conducted experimental evaluations of our proposed method, which combines the ECA module and ICD module, on several datasets: CASIA-SURF [32], CASIA-SURF CeFA [33] cross-racial dataset, CASIA-FASD [14], and OULU-NPU [34].

**Figure 6:**
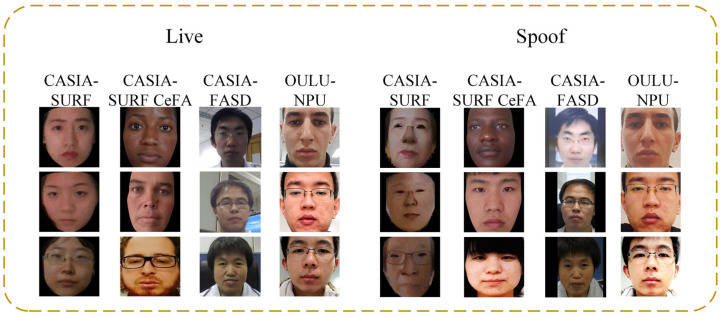
Live and spoofed samples in the CASIA-SURF, CASIA-SURF CeFA, OULU-NPU, CASIA-FASD dataset.

The CASIA-SURF dataset is a large-scale, multi-modal face anti-spoofing dataset that includes RGB, Depth, and IR modalities for each sample, providing rich feature information. As shown in Figure 6, the CASIA-SURF dataset consists of 21,000 videos from 1000 different individuals. Each sample contains six different attack video segments and one genuine video segment. The original video dataset contains a massive number of frames, with the training, validation, and testing sets comprising 1.5 million, 500,000, and 3.1 million frames, respectively. We select one frame every 10 frames and remove data from frames where faces are not detected. After this filtering process, the final training, validation, and testing sets consist of 148,000 frames, 48,000 frames, and 290,000 frames, respectively. The details of the CASIA-SURF dataset are summarized in Table 2.

The CASIA-SURF CeFA cross-racial dataset is a benchmark dataset dedicated to studying racial bias issues in face anti-spoofing. As shown in Figure 6, it provides multimodal data including RGB, depth, and infrared images, covering populations of three different races in Africa, East Asia, and Central Asia, which can help study the performance differences and potential biases of face anti-fraud technology among different races. The CASIA-SURF CeFA dataset includes four protocols to measure different evaluation criteria: Protocol 1 (cross-race), Protocol 2 (cross-PAI), Protocol 3 (cross-modality), and Protocol 4 (cross-race and cross-PAI). We will conduct testing on the three sub protocols of the most challenging protocol 4, as shown in Table 3. The CASIA-SURF CeFA dataset includes a training set, a validation set, and a testing set, with each dataset containing 200, 100, and 200 people of different races.

The CASIA-FASD dataset is a small-scale face liveness detection dataset that includes a variety of spoof and live samples, supporting model training and validation. Its structural design allows the model to be optimized for specific attack methods, thereby improving detection accuracy and resistance to interference. As shown in Figure 6, the CASIA-FASD dataset consists of 50 genuine subjects, each with 12 video clips (3 real videos and 9 attack videos), resulting in a total of 600 video segments. It includes multiple liveness detection methods, such as photo print, video replay, and 3D face detection. The detailed information of the CASIA-FASD dataset is provided in Table 4.

The OULU-NPU dataset is a benchmark dataset specifically designed for face anti-spoofing detection. It provides samples from various attack types and includes different lighting and background conditions, which helps to enhance the model’s performance in real-world applications, as shown in Figure 6. As detailed in Table 5, the dataset consists of 4950 video clips from 55 subjects, covering four different attack types (such as print attacks, video replay, etc.) and filmed under six different environmental conditions. The dataset offers four evaluation protocols: Protocol 1 (impact of lighting variation), Protocol 2 (impact of PAI variation), Protocol 3 (impact of camera device variation), and Protocol 4 (impact of lighting, PAI, and camera device variation). We evaluate the model’s generalization capability using these four protocols.

### 4.2. Metric

The five basic metrics used in this experiment are True Positive (TP), False Positive (FP), True Negative (TN), False Negative (FN), and Equal Error Rate (EER). These metrics are used to calculate the Normal Presentation Classification Error Rate (NPCER), Attack Presentation Classification Error Rate (APCER), Average Classification Error Rate (ACER), and Equal Error Rate (EER). The EER is used to evaluate the model’s performance on the CASIA-FASD dataset, where EER represents the error rate at which the False Acceptance Rate (FAR) equals the False Rejection Rate (FRR). The APCER is expressed as

(15) $${APCER}_{PAIS}=FAR=1-(\frac{1}{N_{PAIS}})\sum_{i=1}^{N_{PAIS}}{Res}_{i}$$

In Equation (15), PAIS is the provided face sample, and *N_PAIS_* represents the frequency of times the sample has been attacked. If sample *i* is classified as a spoof face, Res_i_ is marked as 1; if judged as a genuine face, *Res_i_* is marked as 0. The NPCER is expressed as

(16) $$NPCER=ERR=(\frac{\sum_{i=1}^{N_{BF}}{Res}_{i}}{N_{BF}})$$

In Equation (16), *N_BF_* represents the number of genuine samples. If the *j* sample is an attacked face, *Res_i_* is equal to 1; otherwise, *Res_i_* is equal to 0. The ACER is expressed as

(17) $$ACER=\frac{{APCER}_{PAIS}+NPCER}{2}$$

Equation (17) represents the Average Classification Error Rate, which shows the algorithm’s overall error rate for positive and negative samples. The smaller the ACER value, the better the generalization performance of the algorithm.

### 4.3. Implementation Details

We use PyTorch 1.11.0 to build a deep learning framework. We enhance the data by flipping, rotating, and cropping the face images, and adjust the size of the face images to 112 × 112. Training was conducted on an NVIDIA RTX 4090D GPU, using a stochastic gradient descent (SGD) optimizer. Considering convergence speed and model stability, the learning rate for fine-tuning the pre-trained model is selected from {1 × 10^−3^, 1 × 10^−4^, 1 × 10^−5^} using grid search to determine the optimal convergence rate. To balance computational resources and training efficiency, the batch size is fine-tuned within {64, 128}, with the training process running for 10 rounds, each consisting of 50 epochs. The weight decay is set to 0.005. For momentum decay, the fine-tuning range is {0.5, 0.7, 0.9}, with the optimal value selected through hyperparameter search. Additionally, the loss weight coefficients α, β, λ, γ are chosen from {0.3, 0.5, 0.8, 1}, with the best-performing combination determined through experimental tuning.

In addition, real-time face anti-counterfeiting is crucial for practical applications, and its deployment on real-time devices is necessary. In this study, we proposed a model with an average inference time (FPS) of 46 frames, which can meet most real-time processing requirements and achieve a good balance between performance and efficiency.

## 5. Results Analysis

### 5.1. Comparison Results

#### 5.1.1. CASIA-SURF Dataset

On the CASIA-SURF dataset, we used image patches of different sizes (32 × 32, 48 × 48, and full image) as inputs and compared the performance of our method with models such as ResNeXt-50 [35], Large-Scale Multimodal [32], Spatial Pyramid Pooling [36], Spatial and Channel Attention [37], TTN-S [38], and ResNeXt-SE [39]. The experimental results are presented in Table 6. Our proposed method achieved the best performance across all input sizes, with the lowest ACER of 2.45% when using the 48 × 48 size. This indicates that this size strikes an optimal balance between resolution and computational complexity, effectively capturing detailed image features while avoiding the redundancy introduced by larger sizes. Additionally, when using the full image input, the model also demonstrated significant performance improvement, reflecting its ability to process a wide range of contextual features. Although the ACER for the 32 × 32 size was 2.82%, which still performed well, it was slightly inferior to that of the 48 × 48 input, suggesting that excessively small inputs limit the model’s ability to capture fine-grained details. In conclusion, selecting an appropriate image size is crucial for model performance, as the right size achieves an optimal trade-off between detailed feature extraction and computational efficiency.

#### 5.1.2. CASIA-SURF CeFA Dataset

On the CASIA-SURF CeFA dataset, under Protocol 4 (which combines cross-racial and cross-PAI attacks), we tested three sub-protocols: Protocol 4_1, Protocol 4_2, and Protocol 4_3, and compared the performance with methods such as PSMM-Net [33], 3D-ResNet, SD-Net, ResNet_face [40], and CDCN [41]. As shown in Table 7, the experimental results indicate that our method achieved leading performance with ACER values of 4.3%, 2.78%, and 1.16% for Protocols 4_1, 4_2, and 4_3. In Protocol 4_1, the training and validation sets consist of Africa, while the test sets are from Central Asia and East Asia. The experimental results are slightly worse, which may be due to significant differences in skin color and facial features between Africa and Central Asia and East Asia, resulting in lower adaptability of the model in cross-racial testing; In contrast, Protocol 4_2 and Protocol 4_3 use Central Asia and East Asia as their respective train and validation sets, with test sets containing either East Asia or Central Asia. This suggests that Central Asia and East Asia share more similar face structures and skin tones, allowing the model to achieve better generalization performance.

#### 5.1.3. CASIA-FASD Dataset

Table 8 presents a comparative analysis of our method against existing approaches on various evaluation metrics in the CASIA-FASD dataset. Our proposed method demonstrates exceptional performance in the RGB-based classification task. Specifically, our method achieved an ACER of 1.74% and an EER of 2.27%, significantly outperforming other methods.

#### 5.1.4. OULU-NPU Dataset

The internal testing results on the OULU-NPU dataset are shown in Table 9. Except for Protocol 1, our method outperforms others in Protocols 2, 3, and 4. In Protocol 1, our method achieves an ACER of 1.5%, slightly lower than the best-performing approach. Protocol 1 primarily evaluates model performance under varying lighting conditions. The slight performance drop in this protocol may be due to the model’s limited sensitivity to spoofing samples under extreme lighting conditions. For example, drastic lighting variations can make it challenging to capture subtle face feature differences, affecting the distinction between live and spoofed samples. Additionally, certain attack types may be more deceptive in low-light or high-brightness environments. In contrast, our method achieves outstanding results in Protocols 2, 3, and 4, demonstrating strong stability when handling variations in PAI, camera devices, and the combined impact of lighting, PAI, and camera changes. This further validates the robustness of our approach in complex scenarios with multiple interfering factors. In future research, we aim to further optimize the model’s adaptability to lighting variations.

### 5.2. Ablation Analysis

To validate the effectiveness of the ECA network structure and ICD feature constraint, we conducted ablation experiments on multiple network architectures, including ResNeXt-50, MobileNet, EfficientNet, and Xception Net, across the CASIA-SURF, CASIA-FASD, and OULU-NPU Protocol 1 datasets. These experiments were performed on various input image sizes to thoroughly assess the efficacy of the ECA and ICD modules. The experimental results are presented in Table 10, Table 11 and Table 12.

#### 5.2.1. The Impact of the ECA Module

To demonstrate the impact of the ECA module on model performance, we compared the performance of four networks: ResNeXt-50, MobileNet, EfficientNet, and Xception Net. As shown in Table 10, Table 11 and Table 12, after incorporating the ECA module, the model’s detection performance showed significant improvement across different datasets and input sizes.

On the CASIA-SURF, CASIA-FASD, and OULU-NPU datasets, when using different patch sizes (32 × 32, 48 × 48, and Full image), the ACER of the ResNeXt-50+ECA network significantly decreases compared to ResNeXt-50. Specifically, with the addition of the ECA module, the ACER values under different patch sizes decrease to 3.26%, 2.77%, and 3.04% on CASIA-SURF, 2.23%, 2.12%, and 2.35% on CASIA-FASD, and 2.24%, 1.71%, and 5.27% on OULU-NPU. These results demonstrate the independent performance enhancement brought by the ECA module. Compared with networks such as MobileNet, EfficientNet, and Xception Net, the network with the ECA module achieves superior performance. In contrast, lightweight networks like MobileNet and EfficientNet have limitations in feature extraction precision, while Xception Net, despite enhancing global feature representation, lacks sufficient attention to local details. In liveness detection tasks, local texture and fine-grained features are crucial. The introduction of the ECA module helps capture these critical features, thereby improving the model’s ability to distinguish between live and spoofed samples.

The above experimental results indicate that the ECA module significantly improves the model’s ability to distinguish live faces and effectively resists various deception attacks.

#### 5.2.2. The Impact of the ICD Module

To investigate the effectiveness of our proposed ICD feature constraints, we compared the performance of four networks: ResNeXt-50, MobileNet, EfficientNet, and Xception Net. As shown in Table 10, Table 11 and Table 12, when the patch size is 32 × 32 and the full image is used, the performance of the model with the ICD module is not significantly different from that of other networks, and even slightly inferior on some datasets. When the patch size is 48 × 48, the model with the ICD module has relatively better performance, significantly surpassing other networks and exhibiting the best performance.

Different patch sizes have a significant impact on model performance. When the patch size is 32 × 32, the image block only contains local details and cannot reflect the overall features. At this time, the ICD module is difficult to extract high-quality features from when the features are insufficient, which limits its performance. Lightweight networks such as MobileNet perform similarly to ICD modules in this case, as they can effectively handle simple features.

When the patch size is the full image, the image contains too much background information, which may interfere with feature extraction. The ICD module may be affected by background noise when optimizing a large number of features, making it difficult to achieve optimal performance, while networks such as Xception Net may have more advantages in global feature extraction.

When the patch size is 48 × 48, the image block contains sufficient details and reflects contextual relationships, and the network can extract more complete discriminative features. In the three datasets, the ACER decreased to 4.51%, 2.09%, and 1.72%, respectively, which is an improvement compared to other network structures.

The above experimental results indicate that the addition of the ICD module enables the model to achieve higher detection accuracy in complex scenes, significantly improving the performance of the model in distinguishing between live and spoofed samples.

### 5.3. Visualization and Analysis

#### 5.3.1. Significance Analysis

To further verify whether the performance improvement of our model compared to the ResNeXt-50 model is statistically significant, we conducted five independent experiments on the CASIA-SURF dataset and recorded the ACER index. We calculated the mean (*μ*), standard deviation (*σ*), and 95% confidence interval using confidence interval analysis based on *t*-distribution. The degree of freedom *df* is 4, and the critical value of the 95% confidence interval is *t*_0_._05,4_ = 2.776. The calculation announcement is

(18) $$\mathrm{CI}=\mu \pm t_{\alpha /2,df}\cdot \frac{\sigma }{\sqrt{n}}$$

As shown in Table 13, the statistical significance results are presented. Our proposed model is significantly better than the ResNeXt-50 model in terms of ACER indicators. According to statistical principles, the two sets of mean confidence intervals do not overlap. Therefore, the null hypothesis is rejected at the a = 0.05 level (*p* < 0.05), indicating that the performance difference is statistically significant.

#### 5.3.2. t-SNE Visualization

In order to more intuitively demonstrate the performance of our model in distinguishing between live and spoofed samples, we conducted visual analysis based on the t-SNE [52] method.

**Figure 7:**
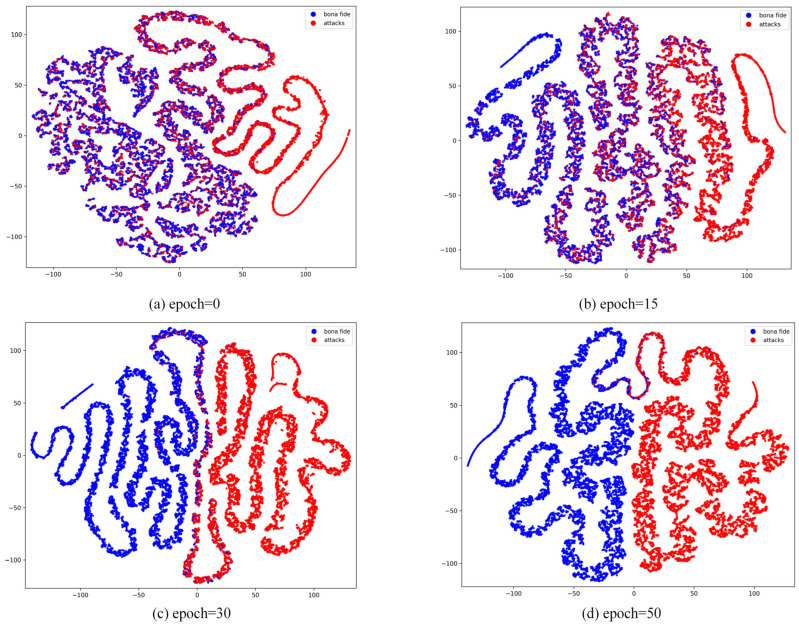
Visualization of t-SNE features between live and spoofed samples during different training periods in the CASIA-SURF dataset.

As shown in Figure 7, we observed the trend of feature extraction and category differentiation during the model learning process by visualizing the feature distribution of the model at different training periods (epoch = 0, 15, 30, 50). At epoch = 0, when the model is untrained, the feature distributions of live and spoofed samples are entirely chaotic, exhibiting no discriminative characteristics. This stage highlights the limitations of feature extraction in a randomly initialized model. By epoch = 15, some features begin to form preliminary clusters, but the distributions of live and spoofed samples still exhibit significant overlap, indicating that while the model has started learning, the classification boundaries remain indistinct, and some samples are not effectively differentiated. At epoch = 30, the feature distributions become more distinct, with two clear clusters emerging for each class, although minor overlaps persist, particularly for samples with complex attributes or less prominent features. This may occur because certain spoofed samples share similar local features with live samples, causing some confusion in the boundary regions. By epoch = 50, the feature distributions stabilize, with live and spoofed samples forming well-separated clusters, demonstrating significant clustering effectiveness. At this stage, the model has effectively learned the key features necessary to distinguish between live and spoofed samples. However, it is worth noting that the minor overlaps in certain extreme cases may stem from the limited distributional differences between live and spoofed samples in the dataset. This analysis demonstrates that the model’s discriminative capability improves significantly as training progresses, leading to a gradual optimization of classification performance.

**Figure 8:**
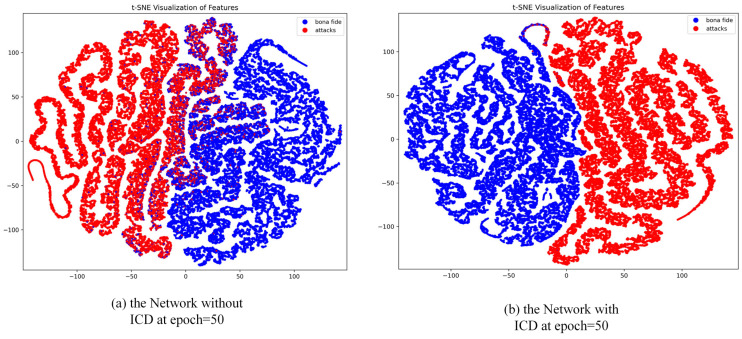
Visualization of t-SNE features between live and spoofed samples in the CASIA-SURF dataset without and with the ICD module.

To further validate the enhancement effect of the ICD module on the model’s feature learning capability, we conducted a visual analysis of the feature distributions at epoch = 50, comparing scenarios with and without the ICD module, as illustrated in Figure 8. Without the ICD module, the feature distributions exhibit some degree of class separation, but significant overlap remains between live and spoofed samples, particularly in cases involving complex sample attributes or highly deceptive spoofing attacks. The model’s ability to constrain features across different classes in the feature space is insufficient, resulting in suboptimal inter-class separation. In contrast, with the ICD module incorporated, the feature distributions improve markedly, with clearer separation between live and spoofed samples and more compact feature clustering. The ICD module optimizes the distribution of features by reducing the intra-class distances and simultaneously increasing the inter-class margins. This enhancement strengthens the model’s discriminative power, resulting in feature distributions that are more distinctive and robust.

**Figure 9:**
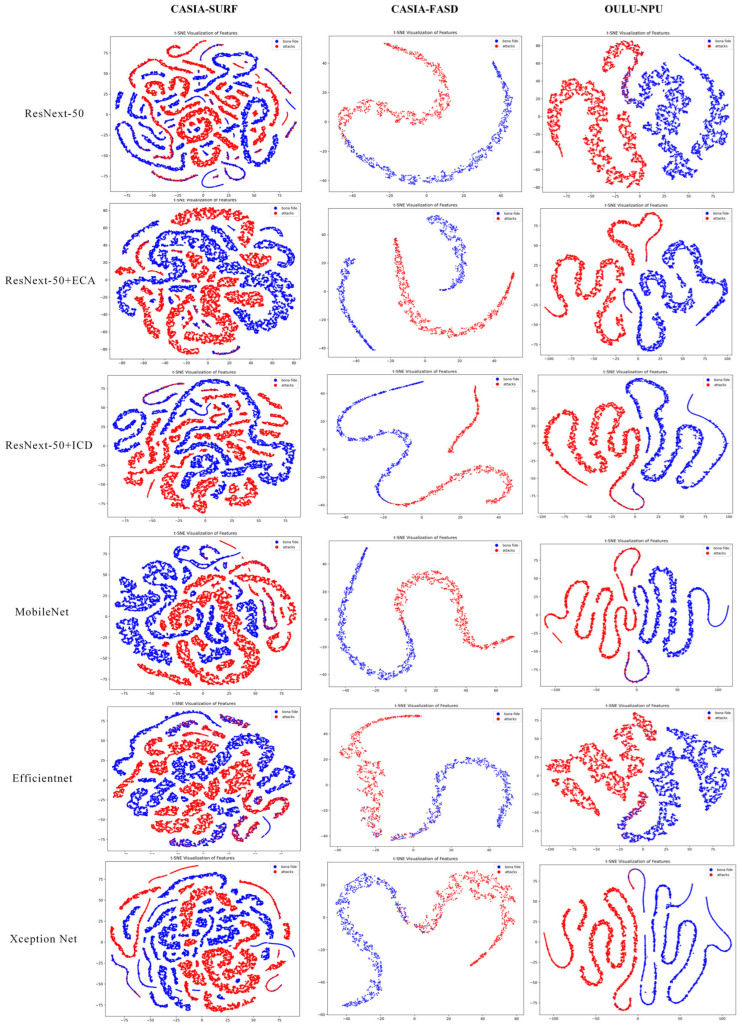
Visualization of t-SNE features between live and spoofed samples for different networks on various datasets with an image size of 48 × 48.

Under the condition of an input image size of 48 × 48, we conducted t-SNE visualization analysis on the features of different networks across the CASIA-SURF, CASIA-FASD, and OULU-NPU datasets. As shown in Figure 9, the incorporation of the ECA and ICD modules results in more compact feature distributions compared to ResNeXt-50, with a clearer distinction between genuine and spoofed samples. The feature distributions of the two classes are distinctly separated into different regions. Although MobileNet, EfficientNet, and Xception Net also demonstrate some degree of discriminative capability, their feature distributions exhibit less compactness and separation compared to our enhanced model. Overall, the ECA and ICD modules improve the model’s feature extraction ability, optimizing feature representation and enhancing classification discriminability, thereby achieving superior detection performance. These visualization results provide intuitive validation of the effectiveness and advantages of our proposed method.

#### 5.3.3. Attention Visualization

To further demonstrate the feature extraction capability of the model, we visualized key regions of live and spoofed samples using the Grad CAM [53] method.

**Figure 10:**
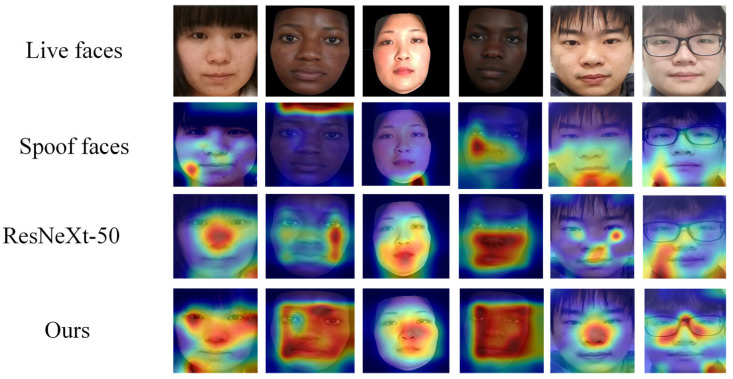
Grad CAM visualization comparison of different models in the CASIA-SURF dataset. Spoof faces cannot form a focus area, while ResNeXt-50 focuses on scattered or background areas. Ours method focuses on the center of the face.

As shown in Figure 10, we compared the Grad CAM visualization results of ResNeXt-50 and our method in the CASIA-SURF dataset to analyze the face regions that the model focuses on and their impact on decision-making. The results indicate that ResNeXt-50 has scattered attention areas and insufficient attention to some key features. On some samples, ResNeXt-50 failed to effectively focus on key face areas, and its heatmap distribution was relatively scattered. In some samples, it even focused on areas unrelated to live discrimination, such as background and edge texture; our method significantly improves the attention and discriminative ability of key areas. By introducing ECA and ICD modules, our method presents a more focused attention area in Grad CAM visualization, especially in the processing of mixed samples. Its heatmap mainly focuses on the core facial feature areas (such as eyes, nose, and mouth), which usually contain rich live information and are most influential in determining live and spoofed samples.

**Figure 11:**
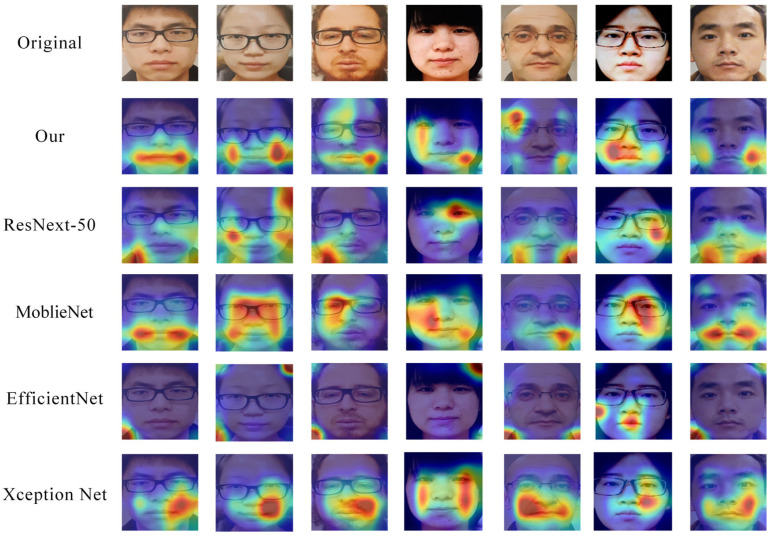
Grad CAM results of different models on visualization failed samples. Ours model focuses on the lips, with ResNext-50 and EfficientNet’s attention scattered around screen borders or face contours. MobileNet and Xception Net’s face attention areas are correspondingly fragmented, without highlighting key areas.

As shown in Figure 11, we present a visual comparison of the networks in the case of failure. Although each network performs differently on these samples, our method can still capture some facial features, but the effect is not ideal, as attention is scattered or focused on non-critical areas. However, ResNeXt-50 showed scattered attention in some samples. The heatmap intensity of EfficientNet is weak, which may result in a random distribution of attention areas due to its lightweight design; MobileNet and Xception Net focus more on certain facial regions and have stronger feature extraction capabilities.

Although our model performs stably in most cases, there are still issues with attention dispersion and insufficient feature extraction when dealing with extreme failure cases such as high light reflection and blurry samples. This results in Grad CAM heatmaps displaying low intensity or attention area shifts, affecting the model’s accurate recognition of spoof features. In addition, when facing failed cases, the model may focus on non-critical areas such as cheeks and forehead, and fail to fully cover key spoof feature areas.

## 6. Conclusions

In this article, we propose an Enhanced Channel Attention (ECA) and Intra-Class Differentiator (ICD) to improve the generalization ability of face detection. The ECA module combines deep convolution and BRMs with the channel attention mechanism, focusing on enhancing the feature expression ability of key channels. The ECA module can extract detailed local and global feature information at different levels, ensuring that the model can focus more on key areas when processing facial features. The ICD module optimizes the sample distribution in the feature space through intra-class compactness constraints and inter-class separability constraints, combined with joint supervision of global local feature comparison loss and cross-entropy loss. The experimental results show that the combination of the ECA module and the ICD module performs well in various test cases, significantly better than the current method.

Although our method performs well across multiple datasets, it still has limitations in extreme scenarios and when encountering unseen attack types. First, under extreme lighting conditions, the channel attention mechanism in the ECA module is susceptible to interference, which may result in insufficient extraction of critical features such as texture details, thereby affecting its discrimination ability. Additionally, low-light conditions may reduce the signal-to-noise ratio, further impacting overall recognition performance. For unseen attack types, the ICD module relies on intra-class compactness constraints to enhance discrimination. However, when faced with entirely new attack methods (e.g., novel 3D masks), this constraint may fail, limiting the model’s generalization ability. Moreover, for samples affected by motion blur or rapid pose variations, the local feature extraction capability of the ECA module may be constrained, leading to attention dispersion toward the background and reducing focus on key face regions. Future work will focus on the following optimizations: 1. Introduce the Vision Transformer (ViT) module to replace traditional convolutional backbone networks and capture cross-regional dependencies through a global attention mechanism to enhance the ECA module’s ability to extract key features under extreme lighting conditions; 2. Use ViT to extract multimodal features, separate attack-irrelevant features through a cross-attention mechanism, and enhance ICD’s discriminative ability against unknown attacks; 3. Combine the ECA module with spatiotemporal ViT such that the ViT layer models inter-frame motion consistency, ECA extracts local details from a single frame, and enhances robustness to motion blur and rapid pose changes through temporal attention fusion. Additionally, we will explore real-time deployment strategies by designing quantization-aware training schemes for edge devices (e.g., smartphones and access control systems) to balance efficiency and accuracy. We believe that with the development of technology, biometric-based security technology will usher in greater breakthroughs. This study provides an effective solution for face liveness detection in complex scenes and lays the foundation for exploring more universal and efficient detection frameworks in the future.

## Figures and Tables

**Table 1:** ECA-ResNeXt Network Architecture.

Layer Name	ECA-ResNeXt	Block
Layer1	Conv 7 × 7.64, stride 2 MaxPool 3 × 3	1
Layer2	Conv1 × 1.128 Conv3 × 3.128, stride 2 Conv1 × 1.256 Dw_conv3 × 3.256 pw_conv 1 × 1.16 pw_conv 1 × 1.256	2
Layer3	Conv1 × 1.256 Conv3 × 3.256, stride 2 Conv1 × 1.512 Dw_conv3 × 3.512 pw_conv 1 × 1.32 pw_conv 1 × 1.512	2
Layer4	Conv1 × 1.512 Conv3 × 3.512, stride 2 Conv1 × 1.1024 Dw_conv3 × 3.1024 pw_conv 1 × 1.64 pw_conv 1 × 1.1024	2
Layer5	Conv1 × 1.1024 Conv3 × 3.1024, stride 2 Conv1 × 1.2048 Dw_conv3 × 3.2048 pw_conv 1 × 1.128 pw_conv 1 × 1.2048	2
	Avg_Pool 7 × 7	

**Table 2:** CASIA-SURF dataset information.

Subset	Subjects	Video	Original Image	Processed Image
Train	300	6300	1,563,919	148,089
Valid	100	2100	501,886	48,789
Test	600	12,600	3,109,985	295,644

**Table 3:** Information on different protocols of the CASIA-SURF CeFA dataset.

Subset	Ethnicity			Subjects	PAIs	Image Number		
	4_1	4_2	4_3			4_1	4_2	4_3
Train	A	C	E	1–200	Replay	33,713	34,367	33,152
Valid	A	C	E	201–300	Replay	17,008	17,693	17,109
Test	C&E	A&E	A&C	301–500	Print	105,457	102,207	103,420

Note: A, C, and E represent Africa, Central Asia, and East Asia; PAIs refer to different attack methods.

**Table 4:** CASIA-FASD dataset information.

Dataset	Resolution	Live Faces	Print Attacks	Cut Attacks	Replay Attacks
CASIA-FASD	480 × 640	8437	9811	9715	7581
	640 × 480	8027	9768	7828	9720
	720 × 1280	10,355	12,435	6687	10,009

**Table 5:** Information on different protocols of the OULU-NPU dataset.

Protocol	Subset	Phones	User	PAIs	Real Videos	Attack Videos	All Videos
Protocol I	Train	6 Phones	1–20	Print; Display	240	960	1200
	Dev	6 Phones	21–35	Print; Display	180	720	900
	Test	6 Phones	36–55	Print; Display	120	480	600
Protocol II	Train	6 Phones	1–20	Print; Display	360	720	1080
	Dev	6 Phones	21–35	Print; Display	270	540	810
	Test	6 Phones	36–55	Print; Display	360	720	1080
Protocol III	Train	6 Phones	1–20	Print; Display	300	1200	1500
	Dev	6 Phones	21–35	Print; Display	225	900	1125
	Test	6 Phones	36–55	Print; Display	60	240	300
Protocol Ⅳ	Train	6 Phones	1–20	Print; Display	200	400	600
	Dev	6 Phones	21–35	Print; Display	150	300	450
	Test	6 Phones	36–55	Print; Display	20	40	60

**Table 6:** Classification results of RGB data in the CASIA-SURF dataset.

Method	Patch Size	APCER (%)	NPCER (%)	ACER (%)
ResNeXt-50 [35]	Full image	21.76	15.06	18.41
Large-scale multimodal [32]	Full image	8.0	14.5	11.3
SPP [36]	Full image	--	--	6.4
Spatial andchannel Attention [37]	Full image	5.2	2.6	3.9
TTN-S [38]	16 × 16	3.8	3.2	3.5
ResNeXt-SE [39]	48 × 48	2.62	2.42	2.52
Our	16 × 16	2.3	3.43	2.87
Our	32 × 32	**2.1**	3.54	2.82
Our	48 × 48	3.84	1.06	**2.45**
Our	Full image	5.57	**0.65**	3.11

Note: Bold values are the best results.

**Table 7:** Classification results of various protocols on the CASIA-SURF CeFA dataset.

Protocol	Method	APCER (%)	NPCER (%)	ACER (%)
Protocol 4_1	PSMM-Net [33]	5.0	3.3	4.2
	3D-ResNet [40]	38.67	5.25	21.96
	SD-Net [40]	5.72	18.5	12.11
	ResNet_face [40]	1.72	12.0	6.86
	CDCN [41]	11.17	2.5	6.83
	Our	7.59	1.16	4.3
Protocol 4_2	PSMM-Net [33]	7.7	9.0	8.4
	3D-ResNet [40]	33.67	6.5	20.08
	SD-Net [40]	7.33	11.25	9.29
	ResNet_face [40]	6.44	12.75	9.6
	CDCN [41]	6.67	2.0	4.33
	Our	3.23	2.33	2.78
Protocol 4_3	PSMM-Net [33]	10.8	4.3	7.6
	3D-ResNet [40]	27.17	6.5	16.83
	SD-Net [40]	3.17	27.0	15.08
	ResNet_face [40]	6.06	16.75	11.4
	CDCN [41]	3.72	3.0	3.36
	Our	**1.21**	**1.12**	**1.16**

Note: Bold values are the best results.

**Table 8:** Classification results of RGB data in CASIA-FASD dataset.

Method	ACER (%)	EER (%)
Fourier-based method [42]	31.24	19.41
CNN [43]	7.31	4.88
DPCNN [44]	6.10	2.90
Patch- and Depth-Based CNN [45]	2.27	2.67
CNN and SWLD [42]	2.14	2.62
**Ours**	**1.74**	**2.27**

Note: Bold values are the best results.

**Table 9:** Internal test results of four protocols in the OULU-NPU dataset.

Protocol	Method	APCER (%)	NPCER (%)	ACER (%)
Protocol 1	GRADIANT [43]	1.3	12.5	6.9
	Auxiliary [46]	1.6	1.6	1.6
	FaceDs [47]	1.2	1.7	1.5
	DeepPixBis [48]	0.8	0.0	**0.4**
	**Ours**	0.64	2.37	1.5
Protocol 2	GRADIANT [43]	3.1	1.9	2.5
	Auxiliary [46]	2.7	2.7	2.7
	FaceDs [47]	4.2	4.4	4.3
	DeepPixBis [48]	11.4	0.6	6.0
	**Ours**	2.67	1.67	**2.17**
Protocol 3	GRADIANT [43]	2.6 ± 3.9	5.0 ± 5.3	3.8 ± 2.4
	Auxiliary [46]	2.7 ± 1.3	3.1 ± 1.7	2.9 ± 1.5
	FaceDs [47]	4.0 ± 1.8	3.8 ± 1.2	3.6 ± 1.6
	DeepPixBis [48]	11.7 ± 19.6	10.6 ± 14.1	11.1 ± 9.4
	**Ours**	2.43 ± 1.19	2.86 ± 2.0	**2.65 ± 1.23**
Protocol 4	GRADIANT [43]	5.0 ± 4.5	15.0 ± 7.1	10.0 ± 5.0
	Auxiliary [46]	9.3 ± 5.6	10.4 ± 6.0	9.5 ± 6.0
	FaceDs [47]	1.2 ± 6.3	6.1 ± 5.1	5.6 ± 5.7
	DeepPixBis [48]	36.7 ± 29.7	13.3 ± 14.1	25.0 ± 12.7
	**Ours**	3.13 ± 4.31	6.81 ± 13.54	**5.05 ± 7.07**

Note: Bold values are the best results.

**Table 10:** Experimental results of various image sizes in the CASIA-SURF dataset.

Patch Size	Method	APCER (%)	NPCER (%)	ACER (%)
32 × 32	ResNeXt-50 [24]	7.19	2.16	4.90
	ResNeXt-50+ECA	2.55	3.97	3.26
	ResNeXt-50+ICD	6.35	2.19	4.27
	MobileNet [49]	3.63	4.31	3.97
	Efficientnet [50]	5.51	4.07	4.79
	Xception Net [51]	4.36	4.96	4.66
48 × 48	ResNeXt-50 [24]	6.97	2.47	4.72
	ResNeXt-50+ECA	3.64	1.9	2.77
	ResNeXt-50+ICD	6.56	2.46	4.51
	MobileNet [49]	6.06	2.17	4.12
	Efficientnet [50]	2.2	7.27	4.73
	Xception Net [51]	2.92	5.92	4.41
Full image	ResNeXt-50 [24]	12.46	11.92	12.19
	ResNeXt-50+ECA	3.64	2.44	3.04
	ResNeXt-50+ICD	7.69	3.87	5.78
	MobileNet [49]	6.06	3.17	4.61
	Efficientnet [50]	5.23	3.78	4.56
	Xception Net [51]	4.89	4.12	4.45

**Table 11:** Experimental results of various image sizes in the CASIA-FASD dataset.

Patch Size	Method	APCER (%)	NPCER (%)	ACER (%)
32 × 32	ResNeXt-50 [24]	3.7	2.9	3.3
	ResNeXt-50+ECA	4.05	2.23	2.23
	ResNeXt-50+ICD	6.1	0.29	3.19
	MobileNet [49]	3.12	2.75	2.93
	Efficientnet [50]	2.98	3.21	3.1
	Xception Net [51]	3.45	2.95	3.2
48 × 48	ResNeXt-50 [24]	0.99	3.72	2.35
	ResNeXt-50+ECA	1.54	2.71	2.12
	ResNeXt-50+ICD	1.82	2.37	2.09
	MobileNet [49]	1.87	3.55	2.71
	Efficientnet [50]	2.09	2.82	2.82
	Xception Net [51]	4.01	2.33	3.19
Full image	ResNeXt-50 [24]	2.25	4.73	3.49
	ResNeXt-50+ECA	1.15	3.55	2.35
	ResNeXt-50+ICD	2.42	3.21	2.81
	MobileNet [49]	1.43	3.72	2.57
	Efficientnet [50]	1.76	3.55	2.65
	Xception Net [51]	1.37	3.89	2.63

**Table 12:** Experimental results of various image sizes in the OULU-NPU dataset.

Patch Size	Method	APCER (%)	NPCER (%)	ACER (%)
32 × 32	ResNeXt-50 [24]	2.34	1.87	2.11
	ResNeXt-50+ECA	2.56	1.92	2.24
	ResNeXt-50+ICD	2.12	2.08	2.1
	MobileNet [49]	2.45	2.15	2.3
	Efficientnet [50]	2.67	1.78	2.23
	Xception Net [51]	2.23	2.34	2.29
48 × 48	ResNeXt-50 [24]	1.83	2.81	2.32
	ResNeXt-50+ECA	2.12	1.31	1.71
	ResNeXt-50+ICD	0.83	2.61	1.72
	MobileNet [49]	2.74	1.31	2.02
	Efficientnet [50]	2.13	2.65	2.39
	Xception Net [51]	1.81	2.15	1.98
Full image	ResNeXt-50 [24]	8.43	3.71	6.07
	ResNeXt-50+ECA	3.02	7.51	5.27
	ResNeXt-50+ICD	3.76	7.6	5.68
	MobileNet [49]	6.28	8.36	7.32
	Efficientnet [50]	8.43	4.47	6.45
	Xception Net [51]	5.34	6.46	5.9

**Table 13:** Summary of model performance significance analysis.

Model	ACER Mean (%)	STD	95% CI
ResNeXt-50	5.218	0.059	[5.145, 5.291]
Our	2.578	0.111	[2.440, 2.716]

Note: STD is the standard deviation, and CI is the confidence interval.

## Data Availability

The data presented in this study are openly available in [CASIA-SURF] at [10.1109/CVPR.2019.00101], [CASIA-SURF CeFA] at [10.1109/WACV48630.2021.00122], [CASIA-FASD] at [10.1109/ICB.2012.6199754] and [OULU-NPU] at [10.1109/FG.2017.77], reference number [14,32,33,34].
